# Evidence for an RNA Polymerization Activity in Axolotl and *Xenopus* Egg Extracts

**DOI:** 10.1371/journal.pone.0014411

**Published:** 2010-12-23

**Authors:** Hélène Pelczar, Anne Woisard, Jean Marc Lemaître, Mohamed Chachou, Yannick Andéol

**Affiliations:** 1 Université Pierre et Marie Curie, UMR 7622, Paris, France; 2 CNRS, UMR 7622, Paris, France; 3 Université Pierre et Marie Curie, Equipe de Photobiologie Moléculaire, Paris, France; 4 Institut de Génomique Fonctionnelle INSERM U661, Montpellier, France; Newcastle University, United Kingdom

## Abstract

We have previously reported a post-transcriptional RNA amplification observed *in vivo* following injection of *in vitro* synthesized transcripts into axolotl oocytes, unfertilized (UFE) or fertilized eggs. To further characterize this phenomenon, low speed extracts (LSE) from axolotl and *Xenopus* UFE were prepared and tested in an RNA polymerization assay. The major conclusions are: i) the amphibian extracts catalyze the incorporation of radioactive ribonucleotide in RNase but not DNase sensitive products showing that these products correspond to RNA; ii) the phenomenon is resistant to α-amanitin, an inhibitor of RNA polymerases II and III and to cordycepin (3′dAMP), but sensitive to cordycepin 5′-triphosphate, an RNA elongation inhibitor, which supports the existence of an RNA polymerase activity different from polymerases II and III; the detection of radiolabelled RNA comigrating at the same length as the exogenous transcript added to the extracts allowed us to show that iii) the RNA polymerization is not a 3′ end labelling and that iv) the radiolabelled RNA is single rather than double stranded. *In vitro* cell-free systems derived from amphibian UFE therefore validate our previous *in vivo* results hypothesizing the existence of an evolutionary conserved enzymatic activity with the properties of an RNA dependent RNA polymerase (RdRp).

## Introduction

Post-transcriptional regulations modulating mRNA stabilities represent important mechanistic steps in the regulation of eukaryotic gene activity. Indeed, it is clearly assumed that half of all changes in the amounts of mRNA is attributed to altered rates of decay [1], [2]. Since 20 years, distinct programs of mRNA decay have been described as post-transcriptional networks controlled by proteins and/or non-coding RNAs which bind to specific sequences or structural elements in the regulated RNAs [3], [4]. Among them, post-transcriptional gene silencing or RNA silencing first discovered in plants and fungi, was reported for a number of animals and protozoa as RNA interference (RNAi) [5], [6]. RNAi is the process whereby double-stranded RNA (dsRNA) induces the homology-dependent degradation of cognate mRNA. This dsRNA is converted into many 21–25 nt short interfering RNA (siRNA) fragments that are further processed by an RNA dependent RNA polymerase (RdRp) activity resulting in the production of secundary siRNA [7], [8]. Soluble recombinant RdRp proteins were purified from tomato and *Neurospora crassa* [9], [10]. They were characterized as RNA template-dependent RNA polymerases *in vitro* and biochemical and genetic experiments have established that RdRp plays a critical role in amplifying the RNAi effect [11], [12]. Up to date, RdRp homolog has only been detected in the genomes of plants, fungi, yeast and nematode [7], [13], [14] but not in the genomes of *Drosophila* and vertebrates. However, post-transcriptional suppression of gene expression by siRNA was established in oocytes and preimplantation embryos of mice [15] as well as in *Xenopus* embryos [16]. A coupling between RNA amplification due to an RNA dependent RNA synthesis and RNA degradation was evidenced in *Drosophila* RNAi where repeated cycles of dsRNA synthesis and concomitant siRNA/primer production result in mRNA degradation [17], [18]. Overall RNAi is conserved in species where no RdRp gene is detected. As a matter of fact, recently, an RdRp activity involved in RNAi and transposon silencing was identified in *Drosophila* [19]. Moreover, an RdRP activity was isolated from human cells and consists of the ribonucleoprotein complex of telomerase reverse transcriptase catalytic subunit (TERT) and of the RNA component of mitochondrial RNA processing endoribonuclease (RMRP) [20].

Using an *in vivo* heterologous system to study RNA stability at the post-transcriptional level, we previously reported a coupling between RNA amplification and accelerated degradation of *in vitro* synthesized *Xenopus* transcripts injected into axolotl oocytes, fertilized or unfertilized (UFE) eggs [21]. After injection, the exogenous RNAs are not continuously degraded over time but are detected with amounts sometimes higher than the injected amounts. The phenomenon occurs with a variety of exogenous sense and antisense substrates. We also established that most of the molecules after injection have the same polarity as the initially injected RNA. Cordycepin 5′-triphosphate prevents increases in RNA levels indicating the involvement of an RNA synthesis [22]. Moreover, we detected the presence of trace amounts of complementary RNA (cRNA), in agreement with the involvement of an RdRp *in vivo* [23]. Similar results were obtained *in vivo* after injection of axolotl RNA into *Xenopus* fertilized eggs, suggesting a phylogenetically conserved mechanism.

In order to further investigate the molecular mechanism of this *in vivo* post-transcriptional process, we intended to use cell-free extracts. In the present study, we develop for the first time low speed extracts (LSE) from axolotl UFE, according to a protocol that minimizes dilution as previously published for *Xenopus laevis* [24]. Such extracts from *Xenopus* UFE have provided the first non viral cell-free system able to faithfully reproduce nuclear synthesis events, from the replication of DNA to the assembly and reconstitution of a functional nucleus [24], [25] and either low- or high- speed extracts can be obtained, depending on the mechanism to be analyzed or the question to be addressed [26], [27]. After characterization of the catalytic properties of the axolotl extracts towards a single stranded M13 DNA dependent DNA polymerization activity, we tested the axolotl and *Xenopus* extracts in an RNA polymerization assay. We show that the amphibian cell-free systems incorporate radioactive ribonucleotide precursors into discrete RNA polymers, indicating that axolotl and *Xenopus* extracts contain all the components necessary to polymerize RNA. When large amounts of an exogenous RNA are added to the extracts, the labelling of a single RNA band similar in size to that of the exogenous RNA is detected. This radioactive labelling does not depend on the free 3′OH extremity of the added RNA and RNA polymerization is not due to terminal labelling of the 3′ end of the RNA. Analysis of the radiolabelled RNA sensitivity to RNase A shows that the RNA produced is single stranded. These results support the hypothesis of an RNA dependent RNA neosynthesis in the axolotl and *Xenopus* mitotic extracts.

## Materials and Methods

### Ethics Statement

The protocol of animal handling and treatment was performed in accordance with the guidelines of the animal ethics committee of the Ministère de l′Agriculture of France.

### Collection of unfertilized eggs (UFE)

Axolotl UFE were obtained from females maintained at 18°C in our laboratory, stimulated 24 h earlier by subcutaneous injection (700 UI) of human chorionic gonadotrophin (hCG; Chorulon, Intervet, France) and kept overnight in 1X HSB (15 mM Tris pH 7.6, 110 mM NaCl, 2 mM KCl, 1 mM MgSO_4_, 0.5 mM Na_2_HPO_4_, 2 mM NaHCO_3_) at room temperature. An axolotl female lays between 800 to 1000 eggs (2.3 mm in diameter). Laid eggs were collected, quickly rinsed with 0.2X HSB brought to pH 8 by addition of NaOH 10N and completely dejellied by gently swirling at room temperature in 3% L-cysteine-hydrochloride, 0.1% papain in 0.2X HSB pH 8. Dejellied eggs were rinsed at least 5 times in 100 mL 0.2X HSB, EGTA 1 mM pH 7.8 per mL of eggs (EGTA : Ethylene Glycol-bis (β-aminoethyl ether)-N,N,N',N'-tetraacetic acid), sorted under a dissecting microscope to remove all damaged or abnormal eggs and eggs were immediately used for preparation of cell-free extracts. *Xenopus* UFE were obtained as previously described [24], [26].

### Preparation of cell-free extracts

Axolotl eggs were first incubated during 5 min in 25 mL cold 1X CSF, 5 mM EGTA buffer extract (2X CSF : 100 mM KCl, 0.1 mM CaCl2, 1 mM MgCl2, 10 mM potassium Hepes pH 7 made by addition of KOH, 50 mM sucrose) then transferred 5 min in 25 mL of 1X CSF, 5 mM EGTA buffer extract containing 10 µg/mL of each protease inhibitors (leupeptin, pepstatin and aprotinin; Sigma). UFE were then transferred in 2.2 mL eppendorfs previously cooled on ice. Excess buffer was removed, leaving only interstitial buffer between packed eggs. After centrifugation at 20 000 g during 50 min (16°C), the cytosolic supernatant was collected, excluding the lipid overlayer, the yolk pellet and the particulate grey interface. The supernatant was collected and Cytochalasin B (10 mg/mL, Sigma) was added to a final concentration of 10 µg/mL. All subsequent manipulations were carried out at 4°C. Cytosolic supernatant was cleared by centrifugation at 20 000 g during 50 min and the soluble phase was collected by puncturing the side of the tube with a 20-gauge needle inserted into a 1 to 5 mL syringue then stored on ice. Protease inhibitors at final concentration of 10 mg/mL each, Energy Mix (1/20 volume) and 5% glycerol were added (Energy Mix 20X : 200 µg/mL Creatine Kinase, 200 mM Creatine Phosphate, 20 mM ATP, 20 mM MgCl_2_, 2 mM EGTA). At this point, Cytochalasin B was added (1 µL at 10 mg/mL per mL of supernatant). After gentle mixing, the clear extracts obtained were stored at −80°C in 50 µL aliquots rapidly frozen in liquid nitrogen. One milliliter of extract was prepared from 500 axolotl UFE. *Xenopus* low speed extracts (LSE) were prepared as previously described [26]. In axolotl and *Xenopus* LSE, the final concentration of non-yolk proteins was determined by Bradford dosage according to Maniatis and Sambrook [28] and estimated to 24 µg or 26 µg for respectively one axolotl or *Xenopus* UFE. These values (25 µg) are in perfect accordance with those found for non yolk proteins in mature *Xenopus* stade VI oocytes [29]. The concentration of total RNA in each extract was determined by LiCl precipitation, phenol/chloroform-chloroform extractions and by absorbance at 260 nm (A_260_) and estimated to respectively 1 and 1.5 µg per one equivalent UFE in axolotl and *Xenopus* extract.

### DNA/RNA template preparations

All the plasmids used as templates in DNA polymerization experiments (single-stranded or double-stranded (7246 base pairs) M13mp18 circular plasmid and double-stranded (2464 base pairs) circular pSP73 plasmid DNA) were purchased at Biolabs as RNase-free grade products and resuspended in the manufacturer's buffer Tris-HCl 10 mM pH 7.5, EDTA 1 mM. Preparation of *Xenopus* demembranated sperm nuclei was as previously described [26]. Capped synthetic RNA templates were generated from linearized plasmids using an *in vitro* transcription kit according to the manufacturer's instructions (Ambion Inc., Austin, TX). The *Xenopus* pGbORFA65 construct was used to synthesize globin RNA [30]. This plasmid contained the 5′UTR, the entire open reading frame and 20 nucleotides of the 3′UTR of the β-globin gene, followed by a poly(A) track of 65 adenines (A). Poly(A)^−^ (without the 65 A; 0.555 kb) and poly(A)+ (with the 65 A; 0.620 kb) sense RNA were obtained by linearization of the plasmid respectively with BamHI or EcoRI (Biolabs) then transcription from T7 promoter of the Bluescript vector. C-myc *Xenopus* RNA was produced *in vitro* using T7 RNA polymerase and the template pXLmyc [31] digested with HindIII to generate the sense 2.2 kb full-lenght transcript. The mouse PKCζ RNA was obtained by linearization of the pBluescriptKS plasmid (gift of S. Louvet) by SalI or XbaI (Biolabs) and transcription from respectively the T3 or T7 promoter of the vector generating sense 534b SalI or antisense 540b transcripts. After the transcription reaction, removal of cDNA template was done by addition of RNase-free DNase (2 U) for 30 min at 37°C. All transcripts were resuspended in water. RNA concentrations were determined by absorbance at 260 nm (A_260_). The ≪standard≫ reaction for addition of cordycepin (3′-deoxy AMP) at the 3′OH ends of *in vitro* transcripts was done according to the poly(A) tailing kit procedure from Ambion (Ref : AM1350) except that cordycepin 5′-triphosphate was used instead of ATP. Briefly, 20 µg of *in vitro* RNA (*Xenopus* globin or c-myc) were incubated with RNase Inhibitor (20 U; Fermentas), 5X poly(A) polymerase buffer, 2.5 mM MnCl_2_, 20 mM cordycepin 5′-triphosphate and *E. Coli* poly(A) polymerase (8 U) in a 100 µL final reaction during 1 hr at 37°C. To check the inability of these 3′OH-modified RNAs to be elongated by any radiolabelled ribonucleotide, 100 µg of *in vitro Xenopus* globin or c-myc transcripts modified at their 3′OH-end (globin 3′H or myc 3′H) or not (globin 3′OH or myc 3′OH) were incubated in a standard tailing reaction (Ambion) containing 1 mM ATP, RNase Inhibitor (30 U), [α–^32^P] rATP (0.3 µM, 10 mCi, Perkin Elmer) and *E. Coli* poly(A) polymerase (12 U) in a final 200 µL reaction during 30 min at 22°C. The size and quality of the different DNA or RNA templates were respectively controlled on a native agarose gel (DNA) or on a formaldehyde gel (RNA) then by electrophoretic migration. RNase A (DNase and protease free) was purchased from Boehringer Mannheim, DNase RQ1 from Promega, α-amanitin, cordycepin and cordycepin 5′-triphosphate from Sigma and RiboRuler RNA ladders from Fermentas.

### DNA and RNA polymerization assays using incubation with amphibian extract followed by TCA precipitation

A ≪standard≫ *in vitro* reaction for a DNA polymerization assay was as follows. Aliquots of frozen extracts were thawed at room temperature immediately before use. To 44 µL of egg extract, 2.5 µL of the 20X ATP-regenerating system (Energy mix) were added to give a final concentration (1X) of 10 mM phosphocreatine (Sigma), 10 mg/mL creatine phosphokinase (Sigma), 1 mM ATP and 1 mM MgCl_2_. Samples were then supplemented with 1 µL of DNA (100 ng of single- or double-stranded DNA or *Xenopus* sperm nuclei equivalent to 25,000 demembranated sperm nuclei, 1 µL of [α–^32^P] dCTP or [α–^32^P] dATP (0.3 µM, 10 mCi) and 0.5 µL of cycloheximide (20 mg/µL, Sigma) or water to obtain a final volume of 50 µL. For a ≪standard≫ *in vitro* RNA polymerization reaction, 2.5 µL of the 20X ATP-regenerating Energy mix system were added to 42 µL of egg extract supplemented with 1 µL RNasin inhibitor (10 U, Invitrogen), 2 µL of RNA *in vitro* template (500 ng), 1 µL (0.3 µM, 10 mCi) of [α–^32^P] CTP or [α–^32^P] UTP and 2 µL containing drugs at the desired concentrations (cordycepin, cordycepin 5′-triphosphate or α-amanitin) to obtain a final volume of 50 µL. For all the reactions, immediately after gentle homogenization, the addition of the radiolabelled precursor defined the t = 0 of the kinetics performed at 20°C, unless otherwise mentionned. At t = 0, five microlitters (1/10 of the initial reaction) was added to 45 µL of ≪Stop mix≫ solution (40 mM EDTA, 1% SDS : Sodium Dodecyl Sulfate) then spotted on a Whatmann GF/C glass filter to determine the total radioactivity of the extract (input). At each time of the kinetics, 1/10 of the reaction was withdrawn and added to 45 µL of ≪Stop mix≫ solution. At the end of the kinetics, each ≪Stop mix≫ solution containing the samples was incubated with 2 µL of Proteinase K (10 mg/mL, Fermentas) during 2 hr at 55°C, then the totality of the reaction was directly spotted on a Whatmann GF/C glass filter. Then filters were TCA (Trichloroacetic Acid) precipitated first at least 3 hr in two batches of cold 5% TCA, 2% PPNA solution (PPNA : tetrasodium diphosphate) (20 mL per filter) and three times two hours in cold 5% TCA then washed twice in ethanol 95%, dried and counting of the incorporated TCA precipitable radioactivity was done in 5 mL of Liquifluor (Amersham). For RNA polymerization assays, an RNase A treatment (Fermentas) was added to the standard procedure : 5 µL of the reaction were incubated with 1 µL of RNase A (10 µg) and water (14 µL) during 1 hr at 37°C.

### DNA analysis, RNA extraction and electrophoretic analyses

For DNA agarose gel analyses, after the polymerization assays using incubations with the amphibian extracts, some samples were treated during 1 hr at 55°C with Proteinase K (10 U/assay), then phenol/chloroform and chloroform (V/V) treated before ethanol precipitation and totally loaded in an 1% agarose native gel before electrophoresis in TEA 1X buffer as described by Maniatis and Sambrook [28]. After transfer to Hybond N^+^ membrane (Amersham, Orsay, France), the filters were washed twice in 2X saline sodium citrate (SSC), pre-hybridized at 65°C in Church buffer [32], hybridized to a radioactive random primed M13 probe at 65°C then washed at this temperature in 0.2X SSC, 0.1% SDS and exposed to a screen before PhosphoImager analysis (Amersham, Molecular Dynamics). For RNA gel analyses, after the polymerization assays, total RNA from each reaction was extracted by the lithium chloride (LiCl) method [33]. Total RNA was then heated 8 min at 65°C and separated by electrophoresis in 1.3% agarose/formaldehyde/MOPS (3-(N-Morpholino) propanesulfonic acid) gels that were first ethidium bromide stained and photographed under UV light. Alternatively, total RNA was separated in 4% acrylamide-bisacrylamide (29 :1) - 8 M urea (TBE 1X) gels that were stained by ≪Stains-all≫ according to the manufacturer (Fluka) and photographed. Agarose and acrylamide gels were then dried and exposed to a screen before PhosphoImager analysis.

### RNA structure analysis

Either total RNA extracted from an RNA polymerization sample using axolotl LSE (48 µl) and an exogenous synthetic transcript (24 µg) or two combinations (10 µg) of *in vitro* transcripts (5 or 10 µg each) were heat-denatured 10 min at 85°C in 80% deionized formamide, 40 mM PIPES pH 6.7, 0.4 M NaCl, 1 mM EDTA (final RNA concentrations : 150 µg/mL). Rehybridization was performed overnight at 65°C. Then the RNA solutions as well as 10X RNase A digestion buffer (3 M NaCL, 0.1 M Tris-HCl pH 8.5, 50 mM EDTA) were diluted 10 fold in a common mix to proceed to RNase A (Fermentas) digestion for 1 hr at 37°C (final RNase A concentrations : 0 ng/mL, 1 ng/mL, 10 ng/mL, 100 ng/mL and 1 µg/mL). Digestions were stopped by incubation with proteinase K (2 mg mL^−1^), 8% SDS during 15 min at 37°C. After phenol/chloroform extraction, RNAs were ethanol precipitated in the presence of glycogen (50 µg/mL; Fermentas) and dissolved in 80% deionized formamide, 50 mM Tris-HCl pH 8.3, 1 mM EDTA, 1% bromophenol blue, 0.1% xylene cyanol. RNAs (corresponding to respectively 1/8 of the RNA polymerization sample or 1/5 of the RNA combinations) were electrophorezed on 8 M-urea 4% polyacrylamide gel for 75 or 30 min at 150 Volts in TBE buffer (0.89 M Tris, 0.88 M Borate, 10 mM EDTA pH 8).

## Results

### Axolotl extracts incorporate radioactive dCMP precursor into DNA in the presence of single-stranded M13 DNA

**Figure 1:**
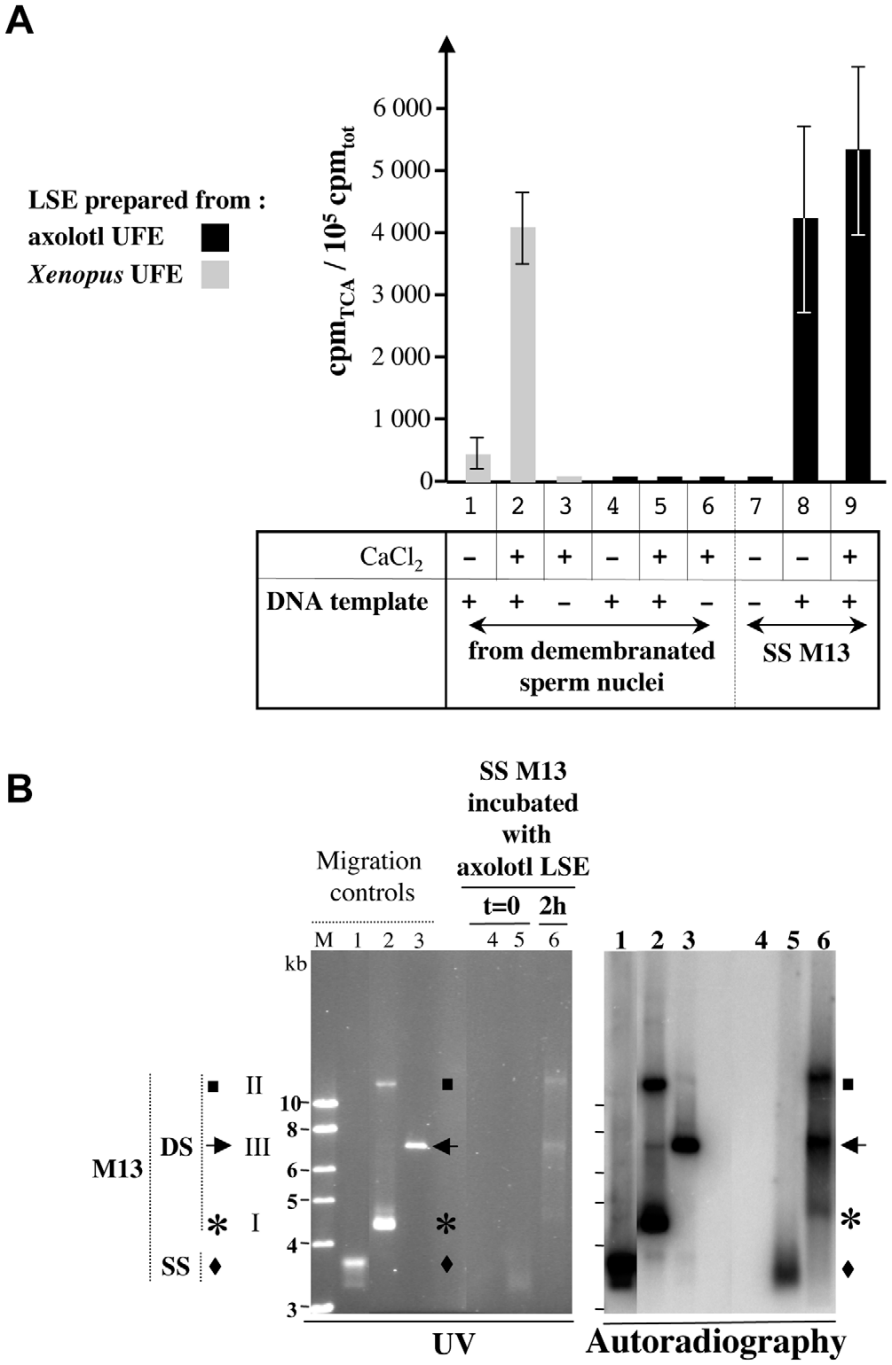
Axolotl low speed extracts (LSE) polymerize DNA when single-stranded M13 DNA is added to the extracts. **A** : Cpm incorporated into TCA precipitable material were registered following 2 hr incubation reactions (44 µL) performed at 20°C in the presence (+) or not (−) of the mentionned DNA template, [α–^32^P] dCTP as DNA precursor, as well as CaCl_2_ addition (+) or not (−). LSE prepared from *Xenopus* UFE and incubated with demembranated sperm nuclei provided the control for dCMP incorporation conditions as well as for activation of LSE by CaCl_2_ addition. Each histogram corresponded to 2 (*Xenopus*) to 5 (axolotl) experiments. **B** : Single-stranded (SS) M13 DNA (150 ng) were mixed in a reaction (88 µL) with axolotl LSE. The reaction was divided into equal aliquots incubated at 20°C during 2 hr (2h, lanes 6) or not (t = 0, lanes 5). A reaction (44 µL, t = 0) without any M13 DNA addition to the extract was also processed (lanes 4). Following phenol/chloroform extractions and ethanol precipitation, the DNA content of each aliquot was analyzed by gel electrophoresis in an 1% agarose gel. Circular M13 DNA either single-stranded (SS; lanes 1 : 100 ng) or double-stranded (DS; lanes 2 : 300 ng) or linearized EcoRI-digested DS M13 (lanes 3 : 400 ng) were electrophorezed in parallel as well as 1 kbp ladder molecular marker (Fermentas, M). Left side : photograph under UV illumination after ethidium bromide staining of the gel. Right side : autoradiograph following gel transfer and hybridization of the corresponding membrane to a radiolabelled SS M13 DNA probe. The migration lengths corresponding to the different forms of M13 DNA are indicated with black diamonds (SS M13), asterisks (supercoiled form of DS M13, I), black squares (closed or nicked relaxed forms of DS M13, II) and black arrows (linearized DS M13, III).

Assuming that it was the first time that axolotl extracts were carried out, we evaluated the quality of this new *in vitro* system according to procedures used for *Xenopus* LSE [26]. We tested the ability of axolotl LSE to catalyze DNA dependent DNA polymerization. Demembranated *Xenopus* sperm nuclei containing 100 ng DNA were incubated with axolotl or *Xenopus* extracts in reactions using [α–^32^P] dCTP as well as Ca^++^ addition or not. As expected, [^32^P] dCMP incorporation into TCA precipitable material was Ca^++^ dependent in *Xenopus* LSE (Fig. 1A, lanes 1–3) [34]. However, no [α–^32^P] incorporation was registered in axolotl LSE (Fig. 1A, lanes 4–6). No incorporation as well was observed with double-stranded (DS) plasmid DNA used as template (data not shown) which has already been reported for *Xenopus* LSE [35]. We then tested the single-stranded (SS) M13 template and figure 1A shows that it is an efficient substrate for dCMP incorporation using axolotl LSE (lanes 7 to 9). When following the incubations, the samples were DNase treated prior to TCA precipitations, no radioactivity was counted above the background defined by a sample where no DNA was added (data not shown). We thus considered that the TCA precipitated material was indeed DNA polymers. Moreover, when visualizing the DNA polymerase reaction products as well as appropriate SS or DS DNA controls in an electrophoresis agarose gel, we observed that following a 2 hr incubation, SS M13 was totally converted into different forms of DS M13 (Fig. 1B).

Overall, these results show that mitotic axolotl LSE represents a successfull eukaryotic system that efficiently converts circular single-stranded M13 DNA to double-stranded DNA.

### Axolotl LSE incorporates radioactive CMP into RNA in the presence or not of exogenous single-stranded RNA

**Figure 2:**
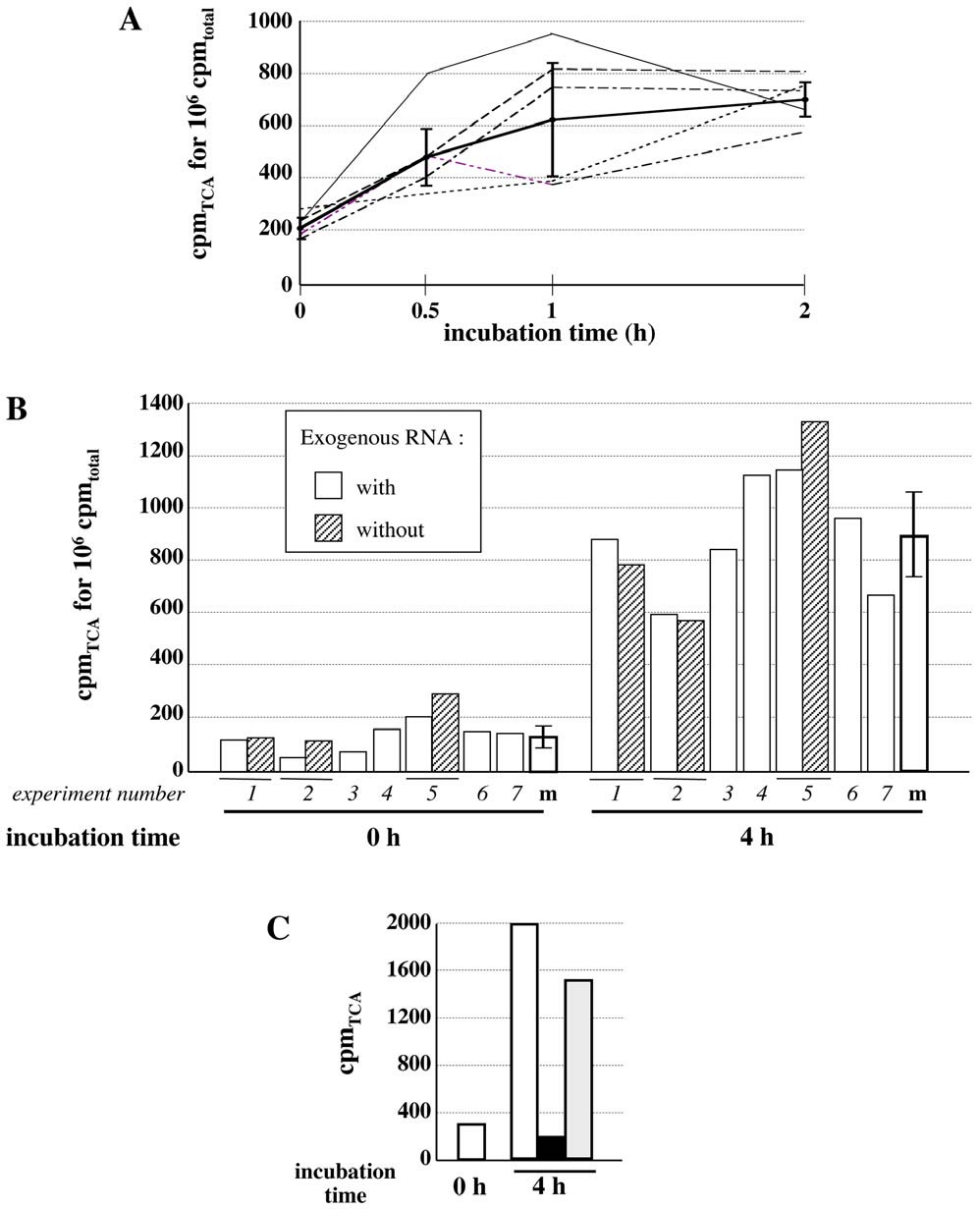
Axolotl extracts incorporate [α–^32^P] CMP into TCA precipitable material. **A**. Time course of incorporation of [α–^32^P] CMP in different axolotl LSEs. Aliquots (5 µL) of a standard reaction (50 µL) using [α–^32^P] CTP were withdrawn throughout kinetics ranging from t = 0 to 2 hours (h), processed for [α–^32^P] CMP incorporation into acid-insoluble material by TCA precipitation and the [α–^32^P] CMP incorporated was expressed as counts per minute (cpm) for 10^6^ cpm used in the reaction (10^6^ cpm_total_). Curves with a thin line corresponded to the kinetics obtained with individual extracts and the mean curve is represented in bold with a standard deviation. **B**. Incorporation of [α–^32^P] CMP in seven independent experiments using the same axolotl LSE. Results obtained using the same LSE during 4 hr-incubation independent experiments (1 to 7) are shown as histograms (white bars) where [α–^32^P] CMP incorporated into TCA precipitable material is expressed as counts per minute (cpm) for 10^6^ cpm used in the reaction (10^6^ cpm_total_). Hatched bars correspond to samples incubated without exogenous RNA and processed in parallel of the standard incubations 1, 2 and 5. White bars surrounded by a bold line (m) represent the mean of the different values, with the corresponding standard deviation, obtained at t = 0 and t = 4 h in incubations with exogenous RNA. **C**. [α–^32^P] CMP is incorporated into RNA. Using an axolotl LSE in a reaction with [α–^32^P] CTP (50 µL), aliquots (10 µL) were collected at t = 0 and t = 4 h. [α–^32^P] CMP incorporated into TCA precipitable material (cpm_TCA_) was determined using a standard protocol (white bars) or a modified one including a further incubation at 37°C with RNase (black bar) or with water (grey bar) prior to TCA precipitation. Similar results were obtained using two other axolotl LSEs.

We then tested the ability of five axolotl extracts to incorporate [α–^32^P] CMP into TCA precipitable material during polymerization assays in the presence of 500 ng of exogenous RNA (Fig. 2). Kinetics over a 2 hr incubation period indicated that [α–^32^P] CMP was indeed incorporated into TCA precipitable material since cpm registered at all the time-points of the kinetics were over the background cpm registered at t = 0, reaching a mean 3 fold above background at 2 hr (Fig. 2A).

Although the amount of incorporated cpm was variable from one point to the other during the kinetics and from one extract to the other, longer incubation times up to 24 hr confirmed [α–^32^P] incorporation with cpm at 6 hr up to 10 fold above the background (data not shown). The use of the same extract in 7 independent experiments (Fig. 2B) did not allow us to reduce the variability in the incorporation rates with a standard deviation corresponding to 30% of the background value and 18% after 4 hr incubation. However, it clearly shows a mean increase of the incorporated cpm at 4 hr consisting in 6.5 fold above the background. Moreover, we observed that addition of 500 ng exogenous RNA had no effect on the total amount of cpm incorporated after 4 hr incubation. To further characterize the reaction products, we introduced a nucleasic treatment following a 4 hr incubation period with the axolotl extract and before the TCA precipitation step. No incorporation at all was detected when samples were treated with RNase A prior to TCA precipitation, indicating that CMP is incorporated into an RNA polymer. This absence of incorporation was not due to the modification of the protocol since the incorporation obtained in a sibling experiment that omitted RNase A addition but included a one hour incubation period at 37°C exhibited only a low decrease in the TCA precipitable cpm (Fig. 2C). Similar results were obtained for *Xenopus* LSE analyzed in parallel (Fig. S1). In summary, although incorporation rates were low, these experiments indicate that axolotl and *Xenopus* extracts contain all the components necessary to catalyze RNA synthesis. We therefore further characterized this RNA synthesis through visualization of the incubation products separated by electrophoresis in a denaturating agarose gel.

### Axolotl LSE incorporates radioactive CMP into discrete RNA polymers without any exogenous template addition

**Figure 3:**
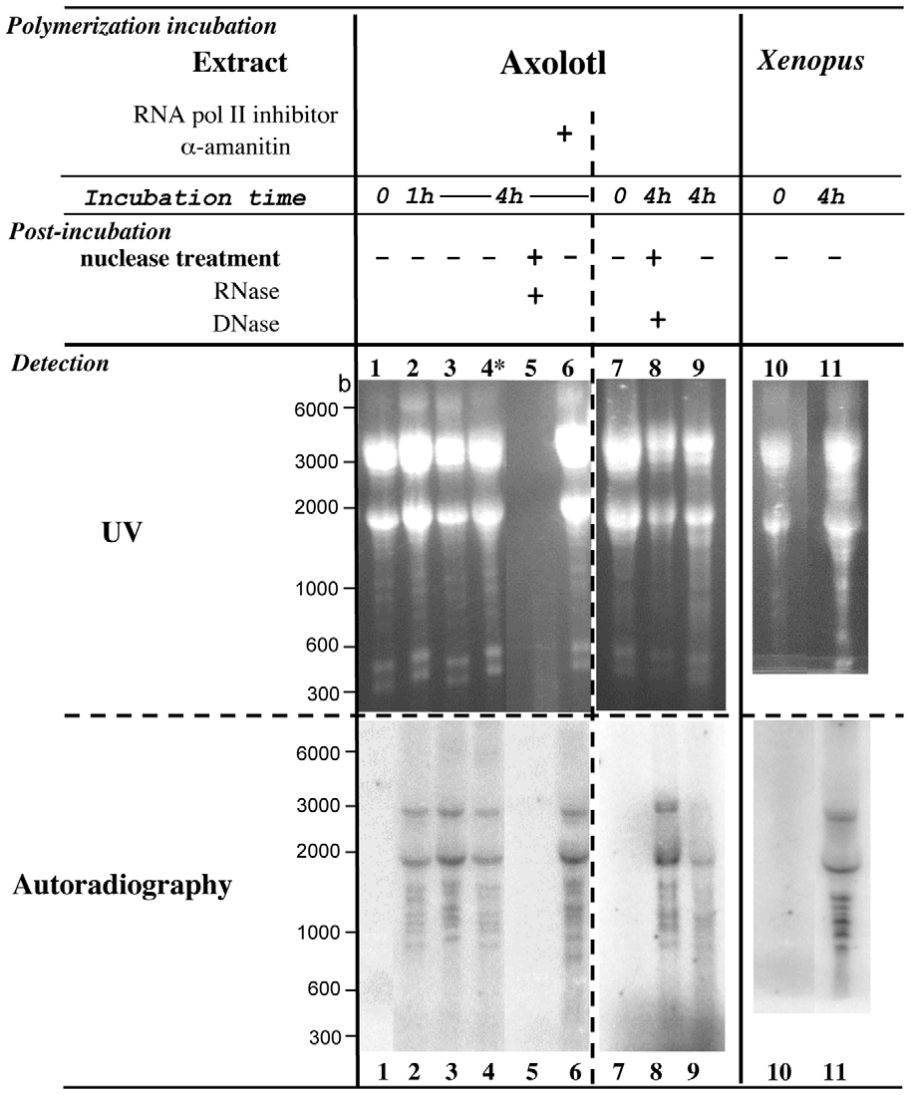
Discrete radiolabelled RNA bands are detected after incubation of the axolotl and *Xenopus* extracts in the presence of [α–^32^P] CTP or [α–^32^P] UTP. Polymerization reactions were performed using axolotl or *Xenopus* LSE with either [α–^32^P] CTP or [α–^32^P] UTP (lane 4) and in the presence (lane 6) or not of 200 µg/mL α-amanitine, an RNA pol II and pol III inhibitor. After the indicated incubation time, samples were treated at 37°C during 1 hr with RNase A (lane 5) or during 15 min with RQ1 DNase (lane 8) or not (lanes 1–4, 6, 7, 9–11). RNA was extracted, electrophorezed through a denaturating agarose gel and visualized after ethidium bromide staining and UV illumination; the gel was dried and autoradiographied. Migration lengths of RNA size markers are indicated in bases (b).

**Figure 4:**
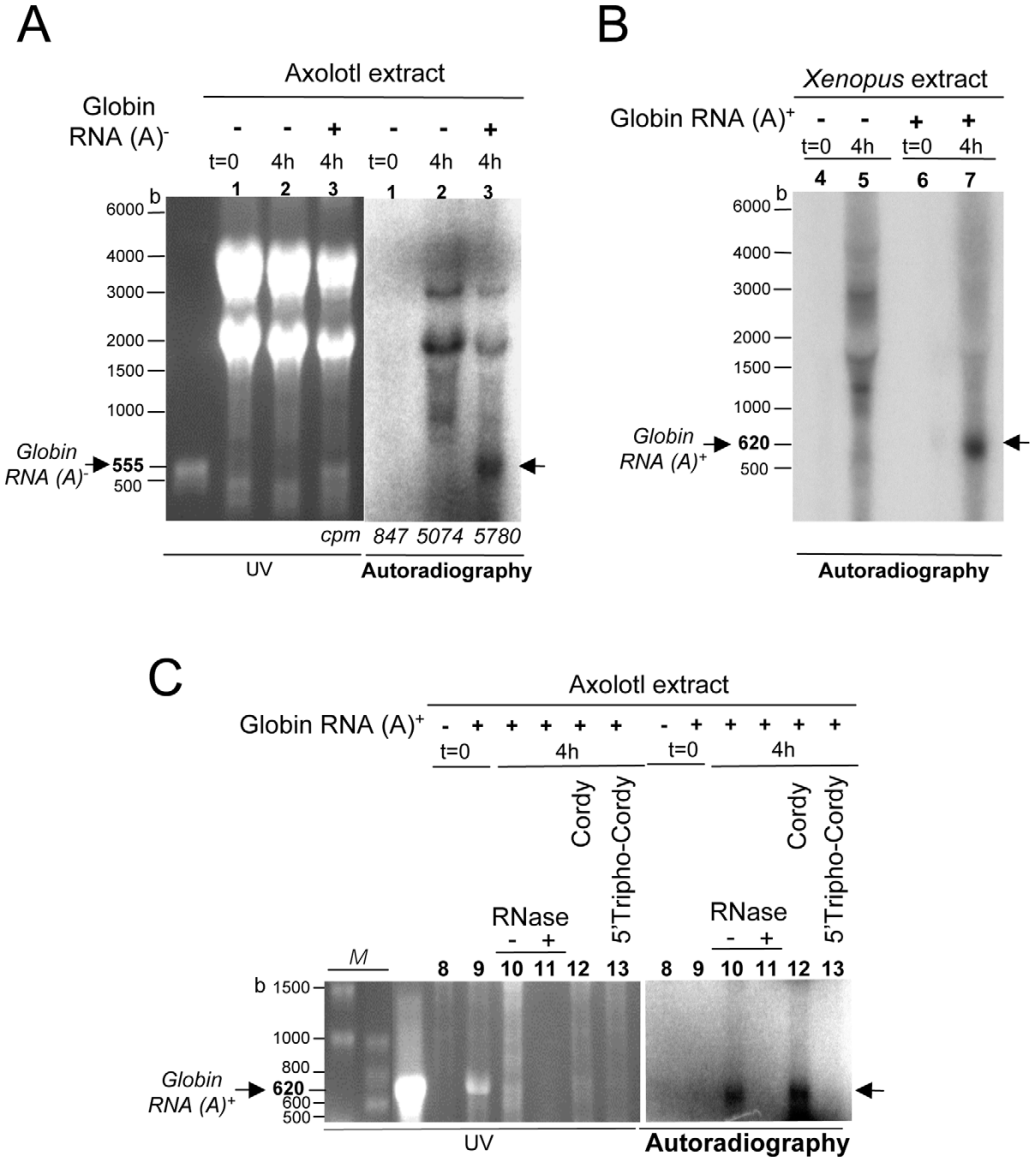
A radiolabelled RNA comigrates with the exogenous RNA added to the extract. Reactions were performed during 4 hr with axolotl (A : lanes 1–3. C : lanes 8–13) or *Xenopus* (B : lanes 4–7) LSE in the presence of [α–^32^P] CTP, in the presence (+) or absence (−) of 10 µg of *in vitro* transcribed *Xenopus* globin 3′UTR poly (A)^−^ (555 b; lanes 1–3 and 8–13) or poly (A)^+^ (620 b; lanes 4–7) RNA and in the presence of 2.5 mM cordycepin (lane 12) or cordycepin 5′-triphosphate (lane 13) or not (lanes 1–11). After incubation, samples were treated at 37°C during 1 hr with 10 mg/mL RNase A (lane 10) or not, RNA was extracted and processed according to the same protocol as in figure 3. The radioactivity corresponding to 1/10 of the reaction analyzed by TCA precipitation is indicated in A (counts per minute : cpm). *In vitro* synthesized *Xenopus* globin 3′UTR poly(A)^−^ (555 b) or poly(A)^+^ (620 b) RNA analyzed in parallel are shown in the UV part of A and C respectively. Migration lengths of RNA size markers (M lanes in C) are indicated in bases (b).

**Figure 5:**
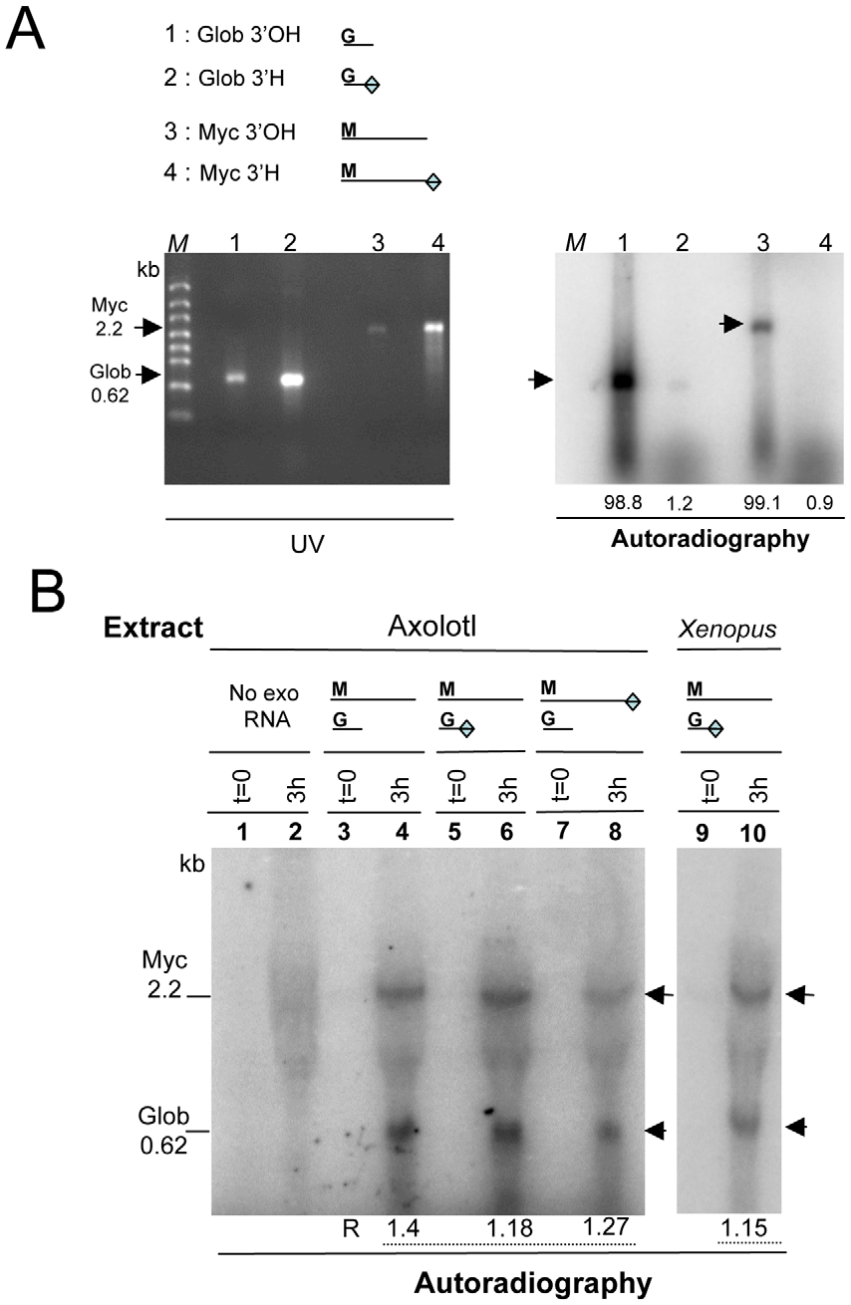
The RNA labelling does not depend on the 3′OH end of the exogenous RNA. **A**. Validation of the modification of the synthetic globin and myc transcripts at their 3′ends. *In vitro Xenopus* globin poly(A)^+^ (lane 1 : 750 ng and lane 2 : 1.2 µg) or myc (Myc, lane 3 : 500 ng and lane 4 : 1 µg) RNA either modified at their 3′OH end by addition of 3′deoxyATP (Glob 3′H, lane 2 or Myc 3′H, lane 4) or unmodified (Glob 3′OH, lane 1 or Myc 3′OH, lane 3) were used in a polyadenylation reaction containing [α–^32^P] ATP, run in an agarose gel stained with ethidium bromide and UV illuminated then immediately dried for autoradiography. The numbers (arbitrary units) indicate that less than 0.5% of the 3′ modified molecules incorporate the radioactive precursor as compared to the corresponding 3′ unmodified synthetic RNA. M  =  RNA molecular Markers High Range ladder. kb : kilobase. **B**. RNA polymerization in amphibian extracts using co-incubation of synthetic globin and myc transcripts with modified or unmodified 3′ ends. Reactions were performed at 20°C during 3 hr (h) with axolotl or *Xenopus* LSE (as indicated), [α–^32^P] CTP and different combinations of exogenous globin and myc *Xenopus* transcripts with 3′OH or 3′H ends (13 µg each, lanes 3–10) except when no exogenous RNA was added (lanes 1–2). After incubation, RNA was processed and analyzed as previously described (Fig. 3 and 4). The ratio (R) of the radioactive signal intensities (Glob/Myc) is indicated for each combination of synthetic RNA. Synthetic transcripts are schematized as in part A. kb : kilobase.

Aliquots of a standard RNA polymerisation reaction without any exogenous RNA were processed at t = 0, t = 1 hr and t = 4 hr by LiCl method then analyzed by agarose gel electrophoresis and autoradiography. As shown in a representative experiment (Fig. 3), low signals were detected following a 4 hr incubation in the presence of radioactive CTP or UTP (lane 4), with two major bands migrating at 1.8–1.9 and 2.7 kb and with 6–7 minor discrete bands ranging from 0.8 to 1.4 kb. In this experiment, the intensity of all bands slightly increased between 1 hr (lane 2) and 4 hr (lane 3). All the signals disappeared after an RNase A treatment (lane 5) but were insensitive to a DNase treatment (lane 8), indicating that these radioactive bands corresponded to RNA. The intensity of the signals detected in the extract incubated in the presence of α-amanitin, a potent inhibitor of RNA polymerases II and III [24], [36], was similar to those obtained without the drug (compare lanes 6 and 3). This suggests that the radioactive labelling corresponds to an enzymatic activity controlled by a mechanism independent of RNA Pol II and III activities. Discrete bands were observed in the *Xenopus* LSE mitotic extract incubated during 4 hr with CTP as radioactive precursor (lanes 10–11). These patterns of discrete bands detected in independent experiments performed from different axolotl and *Xenopus* extracts were not always as clearly observed as in figures 3 and 4 (Fig. 5). However, these results overall confirm that amphibian extracts incorporate ribonucleotide precursors in RNase but not DNase sensitive products, *i.e.* RNA molecules, and indicate that the RNA polymerization is catalyzed by an RNA polymerase activity different from RNA pol II and pol III.

### The addition of an exogenous RNA to axolotl LSE results in the labelling of an RNA similar in size to the exogenous RNA

Interestingly, our preceeding experiment showed that the radiolabelled products were clearly resolved into few discrete bands. One of them corresponding to the 1.8–1.9 kb band comigrates with the 18S rRNA which represents an important endogenous amount of RNA in the extract. This observation led us to study the impact of the addition of a large amount of an exogenous RNA during RNA polymerization with the axolotl extract. Since no radiolabelled bands were detectable with sizes between 2 and 2.7 kb or smaller than 0.8 kb, we choose to design a 0.55 kb and 0.62 kb (Fig. 4) as well as 2.3 kb (Fig. 5) synthetic exogenous RNA for further analysis. We first used ten microgrammes of the *in vitro* synthesized *Xenopus* 3′UTR poly(A)^−^ globin RNA (555 bases). This amount of exogenous RNA was equivalent to 1/4 of the total RNA amount present in an aliquot of 44 µL axolotl extract (35–40 µg). Following a 4 hr incubation, the reaction sample was extracted by LiCl method, then analyzed by ethidium bromide staining/UV illumination and autoradiography. As shown in figure 4A (lane 3), a radioactive signal migrating at the same size as the 555 bases long heterologous RNA was clearly detected. This signal was absent when no exogenous RNA was added to the reaction (lane 2). In this case, the intensity of the signals detected as discrete bands in the axolotl extract appeared higher in absence of exogenous RNA in comparison to the intensity of the same signals detected when the exogenous RNA was present, suggesting a possible competition for the incorporation of the radioactive precursor during the RNA polymerization reaction. Cpm incorporated in TCA precipitable material measured in parallel of gel analysis are in agreement with this hypothesis since they were unchanged upon addition of large amount of exogenous RNA. Moreover, these observations were reproduced when using strongly denaturing 8 M urea-polyacrylamide gels that confirms the sizes of the radiolabelled RNAs detected (data not shown).

Similar results were obtained when 10 µg of the *Xenopus* globin 3′UTR transcript containing a 65 adenosines poly(A) tail at its 3′ end (620 bases) was added either to the *Xenopus* (Fig. 4B, lanes 4 to 7) or to the axolotl (Fig. 4C, lanes 8 to 13) extracts. The signals completely disappeared following an RNase treatment of the reaction products (Fig. 4C, lanes 11) whether they were dependent on the addition of an exogenous RNA or not. Moreover, they were sensitive to cordycepin 5′-triphosphate (*i.e.* 3′-deoxyadenosine 5′-triphosphate, lane 13) an RNA elongation inhibitor, but remained detectable after co-incubations of the transcript with cordycepin (*i.e.* 3′-deoxyadenosine, lane 12) or α-amanitin used at an RNA pol II and pol III inhibiting concentration (200 µg/mL; data not shown). This supports the involvement of cellular enzymes other than RNA pol II and III activities catalyzing the ribonucleotide triphosphate dependent step during precursor incorporation into the radiolabelled RNA polymers.

In summary, addition of large amount of exogenous RNA allows to observe a radiolabelled RNA with the same size as the added RNA. This RNA synthesis appears to occur to the detriment of the RNAs synthesized when no exogenous synthetic transcript is added.

### The radiolabelling does not depend on free 3′OH extremity of the RNA added to the RNA polymerization assay

The preceeding observations were compatible with two hypothesis : elongation of preexisting RNAs from their 3′ ends or neosynthesis of RNA. Since the elongation hypothesis depends on the presence of RNA exhibiting a free 3′OH extremity and since the RNA labelling observed could be provoked by the addition of exogenous RNA, we decided to use exogenous RNA but lacking a free 3′OH extremity. Therefore, a 3′deoxyribonucleotide (cordycepin 5′-triphosphate) was added to the 3′OH extremity of the *in vitro* transcript and we checked that the resulting RNA with a 3′desoxy modified end (3′H end) was unable to incorporate [α–^32^P] ATP in a classical *in vitro* polyadenylation reaction. Synthetic globin and c-myc RNA, with 3′OH or 3′H ends, were incubated *in vitro* in the presence of [α–^32^P] ATP and poly(A) polymerase and further analyzed by electrophoretic migration and autoradiography. As shown in figure 5A, the radioactive labelling detected for the unmodified 0.62 and 2.2 kb RNA (lanes 1 and 3) indicated that our poly(A) tailing conditions were efficient. Therefore the very low labelling detected for the 3′H RNA (lanes 2 and 4; less than 1% of the signal registered for lanes 1 and 3) demonstrated the efficiency of the 3′end modification of these RNAs. When we used RNAs with their 3′ends modified or not in standard RNA polymerization assays with amphibian extracts, we observed radiolabelled RNAs migrating as the exogenous RNAs, whatever the 3′ends of the exogenous RNA used during the incubations (Fig. 5B). This observation therefore supports the RNA neosynthesis hypothesis.

We then compared the intensity of the radioactive labelling detected in axolotl polymerization reactions containing different combinations of exogenous transcripts with 3′OH or 3′H ends. We reasonned that in the neosynthesis hypothesis, an equal amount of each *Xenopus* synthetic globin (0.62 kb) and c-myc (2.2 kb) RNA would result in identical labelling intensities of the 0.62 kb and 2.2 kb radiolabelled signals (ratio = 1) whatever the 3′ ends of the transcripts. However, since an equal amount of each *Xenopus* synthetic RNA corresponds to about 4 fold more globin 0.62 kb transcript as compared to the c-myc 2.2 kb RNA *(i.e*. 4 fold more 3′ globin extremities as compared to 3′ myc extremities), the elongation hypothesis would result in a radiolabelling intensity of the 0.62 kb radioactive signal 4 fold higher than that of the 2.2 kb signal (ratio = 4) if both transcripts harbored 3′OH ends. We first tested the co-incubation of 13 µg of each unmodified *Xenopus* RNA in the axolotl extract in the presence of the radioactive precursor (CTP). As shown in figure 5B (lane 4), the intensity of the radioactive 0.62 kb RNA signal was similar to the 2.2 kb RNA signal, with a ratio around 1.4. Identical ratios were obtained when equal amounts (13 µg) of two exogenous RNAs, one having an unmodified 3′OH end and the other one a modified 3′H end, were incubated during 3 hrs in the axolotl extract (lanes 5–6 and 7–8; ratios : 1.18 and 1.27, respectively) or in the *Xenopus* extract (lanes 9–10; ratio : 1.15). Therefore, these results confirm that the RNA synthesis is not due to a template independent terminal labelling at the 3′OH-end of the exogenous RNA.

### RNA radiolabelled in axolotl extract is single stranded RNA

**Figure 6:**
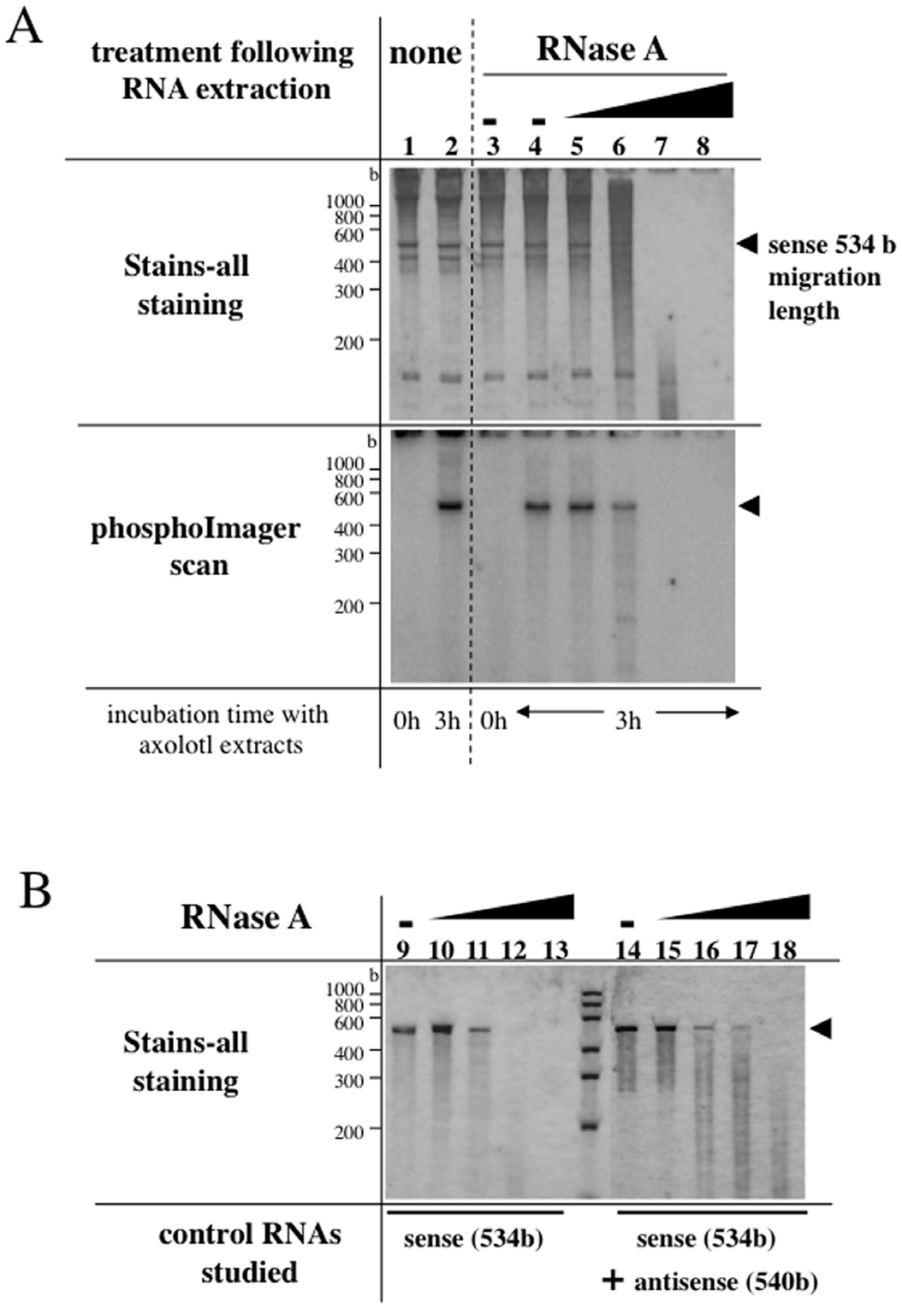
Analysis of the radiolabelled RNA sensitivity to RNase A. RNA polymerization reactions were performed with axolotl LSE and [α–^32^P] CTP in the presence of exogenous sense 534 bases PKCζ synthetic transcript (A); at the indicated incubation times, total RNA was extracted, solubilized in water then either directly mixed with the loading buffer (lanes 1, 2) or diluted in the hybridizing buffer (80% formamide, lanes 3–18). Sense 534 bases PKCζ synthetic transcript was incubated in control hybridization reactions (B), either alone (lanes 9–13) or in the presence of a 540 bases synthetic RNA (antisense) complementary to the sense 534 bases transcript (lanes 14–18). RNAs were heat-denaturated then incubated overnight at 65°C. Following the denaturation/hybridization steps, the ability of the RNAs to resist to RNase A catalyzed hydrolysis was studied in the digestion buffer (8% formamide) and RNase A at final concentrations of 1 ng/mL (lanes 5, 10, 15), 10 ng/mL (lanes 6, 11, 16), 100 ng/mL (lanes 7, 12, 17), 1 µg/mL (lanes 8, 13, 18) or in the absence of RNase A (lanes 3, 4, 9, 14). RNAs were analyzed in polyacrylamide 8 M-urea gels that were stained with ≪Stains-all≫ and exposed to a PhosphoImager screen when mentioned. As compared to the RNase A ≪-≫ conditions (lane 4), the percent of residual radioactivity observed at the migration length of the sense 534 bases transcript (arrow head) has been quantitated : lane 5, 99%; lane 6, 40%; lane 7, 3%; lane 8, 1%. The sizes of the RNA molecular Markers Low Range ladder are indicated in bases (b).

To further investigate the nature of radiolabelled RNAs, we wished to know if the detected RNAs could find a complementary strand in axolotl LSE. We used an experimental approach inspired by classical RNase protection protocols to allow RNA duplex hybridization within the total RNA pool extracted following a 3 hr RNA polymerization reaction. Moreover, we chose to use a large amount of an exogenous synthetic RNA during the RNA polymerization reaction since this favors the synthesis of a radiolabelled RNA at the same size as the exogenous RNA (see Fig. 4). To overcome artefactual secundary structures stabilized during the phenol extraction step, extracted RNAs were heat-denatured and renaturation was performed overnight before RNase A treatments. The range of RNase A concentrations chosen allows to distinguish between single stranded and double stranded RNAs. In this experiment (Fig. 6), the RNA radiolabelled at the end of the incubation with axolotl LSE was expected to be totally hydrolyzed at 100 ng/mL RNase A if, as was observed for the control sense 534 bases RNA alone (lane 12), the radiolabelled RNA is single stranded at the end of the renaturation step. Conversely, the radiolabelled RNA was expected to resist at 100 ng/mL RNase A if it was able to find a complementary strand, like the two reverse complementary single stranded RNAs used as controls (lane 17). The phosphoImager scan clearly shows that the radiolabelled RNA detected at the same migration length as the exogenous RNA used during the RNA polymerization reaction is degraded at 100 ng/mL RNase A (Fig. 6, lane 7). This RNA is therefore single stranded RNA and, in particular, does not form RNA duplexes with the exogenous RNA present in large amount during the hybridization reaction. Accordingly, the long sized radiolabelled RNAs have to be mostly single stranded RNAs and not dsRNAs at the end of the RNA polymerization reaction catalyzed by amphibian extracts.

## Discussion

It is the first time that low speed extracts (LSE) from unfertilized eggs are performed in the urodele axolotl. To characterize the replicating properties of these extracts, classical DNA templates were incubated in polymerization reactions using axolotl LSE and parallel incubations with *Xenopus* LSE as controls. Axolotl LSE is unable to replicate DNA from demembranated *Xenopus* sperm nuclei whether Ca^++^ was added to the incubation mix or not. In a *Xenopus* LSE, incubation of demembranated sperm nuclei leads to the loading of prereplication complexes on DNA and Ca^++^ addition allows the Cdk (cell-cycle dependent kinase) dependent initiation of DNA replication through activation of the licensing factor [37], [38]. Preliminary experiments indicated that axolotl LSE were indeed not responsive to Ca^++^ addition : both the levels of the phosphorylated forms of MAP kinases and Cdk1 (on tyrosine 15) that accumulate in axolotl UFE [39] remained unchanged in axolotl extracts following Ca^++^ addition (data not shown), although they are modulated when intact axolotl UFE are activated [40]. This observation therefore raises the possibility that at least one pathway necessary to the coordinated regulation of Cdk was disrupted during axolotl LSE preparation. We did not prepare extracts from axolotl UFE activated prior to low speed extraction but this would certainly be required to further examine the DNA replicating properties of axolotl extracts.

Considering the molecular requirements for DNA dependent DNA polymerization, it was anticipated that SS M13 DNA would allow DNA polymerization driven by DNA primase and DNA polymerase activities of axolotl LSE. When 150 ng of SS M13 DNA are added to the axolotl extract, the SS M13 DNA is fully converted in DS DNA (Fig. 1B). These results show that one round of polymerization occurs on each input SS DNA and probably only one since DS DNA cannot be used as template by axolotl LSE.

### Evidence for an RNA polymerization that depends on RNA present in amphibian extracts

Our results show for the first time that axolotl and *Xenopus* extracts are intrinsically able to synthesize RNA since addition of a radiolabelled RNA precursor to the extracts allow registration of TCA precipitable cpm that i) are sensitive to 5′-triphosphate cordycepin, an RNA chain elongation inhibitor and that ii) totally disappear following RNase treatment. Previous works showing RNA synthesis in amphibian extracts indeed study transcription and its regulation, *i.e.* DNA dependent RNA synthesis [41], [42], [43]. In these experiments, the RNA polymerase activity that would not depend on the addition of a DNA template is intentionaly kept at a very low detection level. On the contrary, our experiments show that the incorporation rate into RNA polymers observed without addition of an exogenous template to amphibian extracts represents 1% of the input radioactivity following a 6 hr incubation as compared to 0.1% at t = 0 of the kinetics. In the experiments designed to visualize the incubation products, this low incorporation rate requires long exposure times, especially to observe the long sized radiolabelled RNAs (Fig. 3 to 6). In these conditions, the presence of discrete bands ranging from 0.8 to 2.7 kb was detected and we were able to observe an additional band when adding a large amount of exogenous RNA to the extracts. If this indicates that high RNA concentrations are important for the RNA labelling, this is not always true since no radioactivity is detected comigrating with the 28S RNA (4 kb in axolotl) whose amount is equivalent to the 18S RNA, nor with endogenous 350/500 bases RNA whose amount is also high in the extracts (Fig. 3). The RNA synthesis we report is therefore somehow correlated to some but not all the RNAs present at high concentrations in the *in vitro* assay.

### Which enzymatic activity accounts for the RNA labelling?

If *Xenopus* extracts are well known to promote transcriptional repression due to the large store of histones and the resulting assembly of DNA into chromatin [25], [44], pol II and III promoter repression can be released upon histone depletion [25], [44], [45] or TFIIIA addition [43], thereby demonstrating the existence of RNA polymerase II and III activities in amphibian extracts. However, these activities do not probably account for our observations because despite the use of α-amanitin doses known to inhibit them [24], the RNA labelling is still observed.

The detection of radiolabelled RNA comigrating with synthetic or endogenous transcripts present at high concentrations in the amphibian extracts raised the possibility that the RNA synthesis corresponded to the template independent labelling of primer-RNAs at their 3′-ends. This could be due to various enzymatic activities : the first poly(A) polymerase activity identified in vertebrate cells [46] shows a relative fidelity to ATP incorporation in mRNA poly(A) tails but it is also able to incorporate CTP and such an activity exists in the nucleus and cytoplasm of frog oocytes [47]. A polyadenylation activity using CTP and UTP for polymerization of tails at the 3′-ends of ribosomic RNA has been characterized in vertebrates [48]. RNA specific uridylyl transferases are also template-independent polymerases that add ribonucleotides to the 3′ ends of RNA molecules [49]. We tested the 3′ end labelling hypothesis by using large amounts of exogenous RNA harbouring 3′deoxy-ends (Fig. 5). At least for the RNA synthesis dependent on exogenous RNA addition, our results clearly show that the detection of discrete bands does not depend on the 3′OH-end elongation of the exogenous RNA added to the polymerization mix. Moreover, since the labelling of RNA comigrating with exogenous transcripts seems to occur at the expense of the labelling observed when no exogenous RNA is added to amphibian extracts (Fig. 3), we considered that only one enzymatic activity may be involved in the CTP incorporations.

Overall our results therefore suggest that the RNA synthesis does not depend neither on DNA-dependent RNA polymerization activities nor on template-independent 3′OH-end long-sized-RNA extension activities. The RNA labelling could therefore correspond to an RNA template dependent RNA synthesis.

While no homolog of the plants, fungi, *S. pombe* or *C. elegans* RNA dependent RNA polymerases (RdRp) (for a review see [50]) has been identified from the *Drosophila* to the mammalian genomes, cellular proteins harboring RdRp activity have been characterized. In the absence of RdRp encoded by the virus, the RNA templated replication of Hepatitis Delta virus RNA genome depends on cellular enzymatic activities. Several teams have documented RNA dependent RNA synthesis of the antigenomic RNA that is insensitive to α-amanitin as well as RNA dependent RNA synthesis of the subgenomic RNA that is sensitive to the drug, both of them implicating cellular RNA polymerase II associated with a viral protein [51], [52], [53], [54]. Moreover, purified RNA Pol II exhibits RdRp activity whose molecular basis has been shown to reside at the active site used during transcription [55]. Prompted by the need to explain the RNAi in *Drosophila*, a recent work has identified the largest of the three subunits in the RNA polymerase II core elongator complex elp-1/IKAP as an RdRp activity involved in transposon suppression [19]. An RdRp activity was also evidenced for human telomerase reverse transcriptase catalytic subunit (TERT) towards RNA templates that are single stranded transcribed RNA with reverse autopriming properties [20].

In axolotl and *Xenopus* oocytes or UFE, we have previously provided evidence for an *in vivo* RNA polymerization that generates additional copies of RNA and could be part of a general RNA degradation effective during early development [22], [23]. As it has been observed *in vivo*, our *in vitro* results are ascribable to an asymetric RNA synthesis process. Indeed we show here that the radiolabelled RNA with the same size as the exogenous transcript is mostly single stranded, *i.e.* corresponds to RNA of a single polarity. Since the RdRp activities of TERT or elp-1/IKAP produce dsRNA, this cannot explain the RNA polymerization catalyzed in the amphibian LSE. Nevertheless, RNA dependent RNA synthesis is asymetric in viral RNA replication [56] and for RNA amplification of cytoplasmic β-globin RNA in murine erythroleukemia cells [57], [58]. The molecular steps necessary to explain the observations that we present here in amphibian remain to be elucidated. However, since 3′OH-end modification of the added RNA does not preclude the observation of RNA synthesis, none of these steps is strickly dependent on the 3′OH-end of long sized RNAs. In particular, priming of RNA synthesis involving an intramolecular foldback RNA hybridization as a first step can be already excluded.

A difference exists in the effectiveness of the RNA dependent RNA synthesis between the observations we made in amphibian *in vitro* and *in vivo* systems. While it is possible to register *in vivo* a quantitative amplification of the injected RNA, this observation is not reproduced *in vitro* : the intensity of the ≪Stains-all≫ band corresponding to the exogenous transcript is not higher at the t = 3 hr point of the incubation than at t = 0 (Fig. 6). This result suggests that at least one step necessary to RNA synthesis is a limiting step *in vitro*. One protein may have lost its activity during the extraction or could be inhibited by a co-extracted component of the extracts. Optimization of RNA synthesis conditions *in vitro* will be useful to understand the mechanisms that command such RNA polymerization in vertebrates.

## Supporting Information

Figure S1 Xenopus extracts incorporate [α−^32^P] CMP into TCA precipitable material. A. Time course of incorporation of [α−^32^P] CMP in two Xenopus LSEs. Aliquots (5 µL) of a standard reaction (50 µL) using [α−^32^P] CTP were withdrawn throughout kinetics ranging from t = 0 to t = 180 minutes (min), processed for [α−^32^P] CMP incorporation into acid-insoluble material by TCA precipitation and the [α−^32^P] CMP incorporated was expressed as counts per minute (cpm) for 106 cpm used in the reaction (106 cpmtotal). B. Incorporation of [α−^32^P] CMP in four independent experiments using the same Xenopus mitotic LSE. Results obtained using the same LSE during 4 hr incubation independent experiments (1 to 4) are shown as histograms (white bars) where [α−^32^P] CMP incorporated into TCA precipitable material was expressed as counts per minute (cpm) for 106 cpm used in the reaction (106 cpmtotal). Hatched bars correspond to samples incubated without exogenous RNA and processed in parallel of the standard incubation 2. White bars surrounded by a bold line (m) represent the mean of the different values with the corresponding standard deviation obtained at t = 0 h and t = 4 h in incubations with exogenous RNA. C. [α−^32^P] CMP is incorporated into RNA. Using a Xenopus LSE in a reaction (50 µL) with [α−^32^P] CTP, aliquots (10 µL) were collected at t = 0 and t = 4 h. [α−^32^P] CMP incorporated into TCA precipitable material (cpmTCA) was determined using a standard protocol (white bars) or a modified one including a further incubation at 37°C with RNase (black bar) or with RQ1 DNase (grey bar) prior to TCA precipitation. Similar results were obtained using another Xenopus LSE.
(0.40 MB TIF)
