# Supplementary figures and images for: Evidence for an RNA Polymerization Activity in Axolotl and Xenopus Egg Extracts
Source: PLoS One. 2010 Dec 23;5(12):e14411. doi: 10.1371/journal.pone.0014411 (PMC3009717; doi:10.1371/journal.pone.0014411)

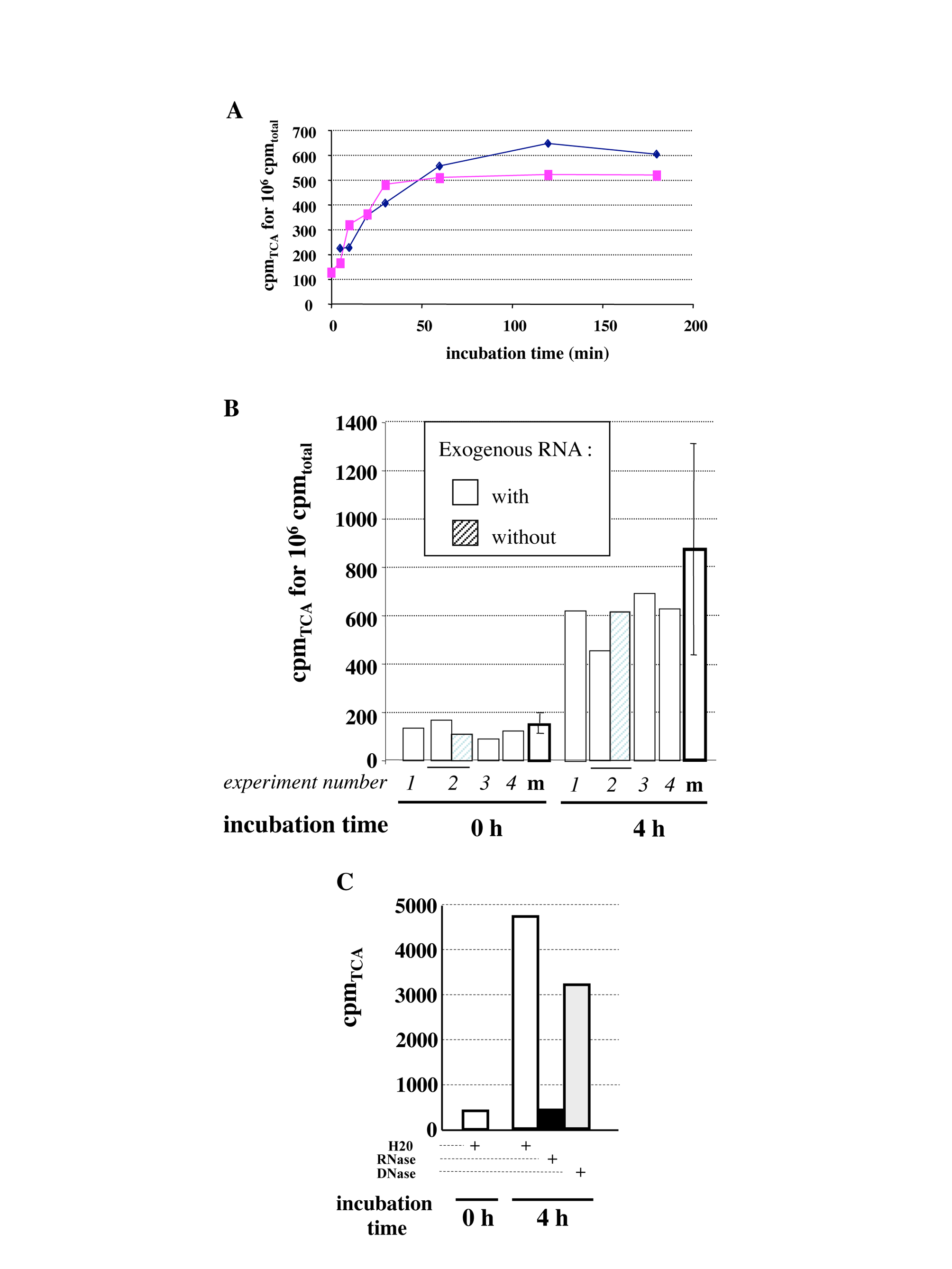

Supplement: Figure S1 — Xenopus extracts incorporate [α−32P] CMP into TCA precipitable material. A. Time course of incorporation of [α−32P] CMP in two Xenopus LSEs. Aliquots (5 µL) of a standard reaction (50 µL) using [α−32P] CTP were withdrawn throughout kinetics ranging from t = 0 to t = 180 minutes (min), processed for [α−32P] CMP incorporation into acid-insoluble material by TCA precipitation and the [α−32P] CMP incorporated was expressed as counts per minute (cpm) for 106 cpm used in the reaction (106 cpmtotal). B. Incorporation of [α−32P] CMP in four independent experiments using the same Xenopus mitotic LSE. Results obtained using the same LSE during 4 hr incubation independent experiments (1 to 4) are shown as histograms (white bars) where [α−32P] CMP incorporated into TCA precipitable material was expressed as counts per minute (cpm) for 106 cpm used in the reaction (106 cpmtotal). Hatched bars correspond to samples incubated without exogenous RNA and processed in parallel of the standard incubation 2. White bars surrounded by a bold line (m) represent the mean of the different values with the corresponding standard deviation obtained at t = 0 h and t = 4 h in incubations with exogenous RNA. C. [α−32P] CMP is incorporated into RNA. Using a Xenopus LSE in a reaction (50 µL) with [α−32P] CTP, aliquots (10 µL) were collected at t = 0 and t = 4 h. [α−32P] CMP incorporated into TCA precipitable material (cpmTCA) was determined using a standard protocol (white bars) or a modified one including a further incubation at 37°C with RNase (black bar) or with RQ1 DNase (grey bar) prior to TCA precipitation. Similar results were obtained using another Xenopus LSE. (0.40 MB TIF) [file pone.0014411.s001.tif]
